# Xiaoqinglong Decoction Enhances Autophagy to Antagonist Airway Inflammation Induced by Cold in Asthmatic Rats

**DOI:** 10.1155/2022/3943343

**Published:** 2022-10-25

**Authors:** Bin Wang, Xiaoxuan Fan, Qianwen Sun, Qingxiang Zhang, Yue Kong, Qingyan Meng, Dongsheng Zhao, Beibei Mao, Yan Liu, Pan Zhao, Lu Zhang, Peizheng Yan

**Affiliations:** ^1^College of Pharmacy, Shandong University of Traditional Chinese Medicine, Jinan, Shandong 250355, China; ^2^College of Traditional Chinese Medicine, Shandong University of Traditional Chinese Medicine, Jinan, Shandong 250355, China

## Abstract

Asthma is a common chronic respiratory disease characterized by wheezing and shortness of breath. Its risk factors include genetic and acquired factors. The acquired factors are closely related to the environment, especially cold conditions. Autophagy plays a regulatory role in asthma. Therefore, we hypothesized that asthma can be controlled by drug intervention at the autophagy level under cold conditions. The Xiaoqinglong decoction (XQLT) was freeze-dried. The compounds in the freeze-dried powder were identified and quantified using reference standards via the high-performance liquid chromatography method. Ovalbumin (OVA)-sensitized rats were subjected to cold stimulation. The effect of cold stimulation on autophagy levels was determined, and it was confirmed that cold stimulation affected autophagy. The effects and mechanisms of XQLT in an asthmatic rat model (OVA-sensitized rats stimulated with cold) were explored. The concentrations of paeoniflorin, liquiritin, trans-cinnamic acid, glycyrrhizic acid, 6-gingerol, schisandrol A, and asarinin in XQLT freeze-dried powder were 14.45, 3.85, 1.03, 3.93, 0.59, 0.24, and 0.091 mg/g, respectively. Cold stimulation is an important cause of asthma. The inflammatory factors in bronchoalveolar lavage fluid and serum were increased in the model group, accompanied by a decline in autophagy level. The treatment with XQLT increased the expression of autophagy genes and decreased the expression of inflammatory factors. Histological studies showed that XQLT improved inflammatory infiltration and collagen fiber deposition in the lungs of rats. XQLT intervention increased autophagy in asthmatic rats. Autophagy plays a role in phagocytosis and reduces the accumulation of abnormal metabolites in the body to reduce airway inflammation and promote asthma recovery.

## 1. Introduction

Asthma, one of the most common heterogeneous noncommunicable diseases, is a chronic inflammatory disease that responds to infection, environmental allergens, and stimulants, leading to occasional and reversible airway contractions [1]. Currently, approximately 334 million individuals are diagnosed with asthma globally [2]. The main symptoms of asthma are airway swelling, increased mucus caused by inflammation, and bronchial spasms caused by compression of the muscles around the airway, resulting in dyspnea [3]. Patients with asthma usually experience recurrent attacks or even deterioration of asthma conditions. In clinical practice, corticosteroids are often used to control asthma, and their side effects are evident, affecting patients' quality of life. Therefore, it is necessary to understand the mechanisms involved in asthma attacks to improve treatment options.

Asthma is characterized by cough, wheezing, chest tightness, and shortness of breath and is mainly caused by an imbalance in Th1-/Th2-mediated immunity, which leads to airway inflammation that triggers a series of pathological reactions. The onset of asthma is related to many factors, including genetic, environmental, and dietary factors. Environmental factors play a major role in an asthma episode, especially in cold conditions [4]; the mortality rate is high in asthmatic adults during winter. Cold stimulation aggravates the condition of patients with asthma, which can cause inflammatory infiltration of the upper respiratory tract and pulmonary bronchial contraction, affect the ventilation function of the airway, and lead to dyspnea [5]. Therefore, it is important to study the pathogenesis of asthma under cold stimulation and intervene in this mechanism to reduce asthma attacks.

Autophagy is a natural phenomenon for cells to maintain a steady state. In this process, autophagy-associated proteins and a series of complexes are adsorbed onto the autophagosome membrane, and proteins and organelles that need to be eluted enter the phagophore through cargo receptors, such as sequestosome 1 (SQSTM1)/p62, on the membrane and ubiquitin marks on autophagic cargo. After fusion with lysosomes, the contents are digested and released into the cytoplasm for energy reuse [6]. Environmental stress activates autophagy by activating the regulator of autophagy. Autophagy is an important mechanism for organisms to survive under various environmental stresses. Selective autophagy can regulate various physiological and pathological conditions [7]. Autophagy can control adaptive immunity in the respiratory system by regulating antigen presentation and lymph node development, directly eliminating pathogens, or regulating cytokine signals to induce innate immunity [6]. Studies have shown a strong association between autophagy and severe asthma [8, 9]. Genomics studies have identified autophagy-related genes; the rs12212740 gene locus of ATG5 is strongly related to the development of asthma, in which the G allele is considered a risk factor for asthma and positively correlated with a decrease in forced expiratory volume in 1 s [10]. Inhibition of autophagy in rats treated with 3-methyladenine or knockdown of ATG5 inhibited the expression of eosinophils and interleukin (IL)-5 in the bronchoalveolar lavage fluid (BALF), thereby alleviating asthma symptoms [11]. Increased expression of double-membrane autophagosomes in epithelial cells and fibroblasts of bronchial tissue is present in patients with moderate and severe asthma, detected by electron microscopy [10]. Autophagy is active in the occurrence and development of bronchial asthma, and the autophagy pathway in airway epithelial cells may be an important mechanism of asthma onset and a potential target for asthma treatment.

Autophagy plays a protective role in pulmonary diseases. Inhibition of autophagy leads to the accumulation of p62. Excessive p62 competes with nuclear factor erythroid 2-related factor 2 (Nrf2) for keap1, and Nrf2 regulates protective genes involved in scavenging reactive oxygen species. p62 destroys the connection between Nrf2 and keap1, thereby reducing the ubiquitination and degradation of Nrf2. Its downstream target is activated, which promotes the clearance of free radicals and reduces the secretion of inflammatory factors [12]. The knockout of autophagy genes (ATG7, ATG5, and ATG4b) or the use of autophagy inhibitors can promote the development of lung injury in mice [13]. Another study evaluating the aging of the airway epithelium and interstitial tissue in human lung specimens found that insufficient autophagy causes the development of fibrosis [14]. These events suggest that the potential pathogenesis of airway hyper-responsiveness might be related to insufficient autophagy. Therefore, the regulation of autophagy may be ideal for the treatment of asthma.

Xiaoqinglong decoction (XQLT) originates from the Treatise on Febrile and Miscellaneous Diseases, which was authored by Zhang Zhongjing in A.D. 205. XQLT is composed of Ephedrae Herba, *Cinnamomum cassia* Presl, Paeoniae Radix Alba, Asari Radix et Rhizoma, Zingiberis Rhizoma, Glycyrrhizae Radix et Rhizoma, Pinelliae Rhizoma, and *Schisandrae chinensis* Fructus. XQLT relieves cough and asthma and is widely used for the treatment of lung and respiratory diseases in China. In a study on cold-induced asthma, XQLT reduced the levels of tumor necrosis factor (TNF)-*α*, IL-1*β*, thymic stromal lymphopoietin (TSLP), and nuclear factor kappa light chain enhancer of activated B cells (NF-*κ*B) in the serum of mice, effectively inhibited the expression of TSLP and NF-*κ*B in lung tissue, and improved the infiltration of lung inflammation, suggesting that the TSLP pathway may be a target of XQLT in treating asthma [15]. Cold is a common factor that induces asthma; therefore, winter is associated with a high incidence of asthma. XQLT is a classic prescription for treating asthma with the cold syndrome, but there are few studies on the detailed mechanism of its effect on asthma and autophagy. Therefore, this study established an asthma model sensitized with ovalbumin (OVA) and stimulated with cold and used XQLT for treatment to verify the effect of XQLT intervention on autophagy in cold-stimulated bronchial asthma combined with relevant detection methods.

## 2. Materials and Methods

### 2.1. Materials and Reagents

The following herbs were assessed in this study: Ephedrae Herba (Menggu, China), *C. cassia* Presl (Guangxi, China), Glycyrrhizae Radix et Rhizoma (Gansu, China), Zingiberis Rhizoma (Yunnan, China), Paeoniae Radix Alba (Anhui, China), Asari Radix et Rhizoma (Liaoning, China), Pinelliae Rhizoma (Shandong, China), and *S. chinensis* Fructus (Liaoning, China). The reference standards 6-gingerol (Lot B21838, purity >98%), schisandrol A (Lot B21322, purity >98%), asarinin (Lot B21346, purity >98%), cinnamic acid (Lot B21082, purity >98%), paeoniflorin (Lot B21148, purity >98%), liquiritin (Lot B20414, purity >98%), and glycyrrhizic acid (Lot B20417, purity >98%) were purchased from Shanghai Yuanye Bio-Technology Co., Ltd. (Shanghai, China).

Acetonitrile of high-performance liquid chromatography (HPLC) grade was purchased from Oceanpak Alexative Chemical Co., Ltd. (Sweden). HPLC-grade phosphoric acid was purchased from Shanghai Aladdin Biochemical Technology Co., Ltd. (Shanghai, China). Analytical grade methanol was purchased from Sinopharm Chemical Reagent Co., Ltd. (Shanghai, China). Other reagents used in this study were ovalbumin (OVA, Lot A8040, Solarbio, Beijing, China) and aluminum hydroxide (Lot 20200226, Sinopharm Chemical Reagent Co., Ltd., Shanghai, China). Rat IL-13 enzyme-linked immunosorbent assay (ELISA) kit (Lot CSB-E07454) and Rat IL-4 ELISA kit (Lot CSB-E04635r-96T) were purchased from Cusabio Biotech Co., Ltd. (Wuhan, China). Rat TNF-*α* ELISA kit (Lot ER006-96) and Rat interferon (IFN)-*γ* ELISA kit (Lot ER005-96) were purchased from ExCell Bio-Technology Co., Ltd. (Taicang, China). The following antibodies were purchased from Abcam (Cambridgeshire, England): antihypoxia-inducible factor 1 (HIF1)-*α* antibody (Lot ab2185), the antimechanistic target of rapamycin (mTOR) recombinant antibody (Y391) (Lot ab32028), anti-mTOR (phospho S2481) recombinant antibody (Lot ab137133), anti-SQSTM1/p62 antibody (Lot ab56416), anti-Beclin 1 antibody (Lot ab62557), and light chain 3 (LC3) A/B (D3U4C). XP rabbit mAb (lot 12741s) was purchased from Cell Signaling Technology, Inc. (Danvers, MA, USA). The Masson dye kit (LotG1006) was purchased from Servicebio Biotechnology Co., Ltd. (Wuhan, China).

### 2.2. Drug Preparation

The compound preparation of XQLT used in this experiment originated from the Treatise on Febrile and Miscellaneous Diseases and consisted of eight herbs. According to the fifth edition of Formulaology, the dosages were as follows: Ephedrae Herba, 9 g; *C. cassia* Presl, 6 g; Paeoniae Radix Alba, 9 g; Asari Radix et Rhizoma, 9 g; Zingiberis Rhizoma, 3 g; Glycyrrhizae Radix et Rhizoma, 6 g; Pinelliae Rhizoma, 9 g; and *S. chinensis* Fructus, 3 g. These dosages are commonly used in clinical settings.

The Ephedrae Herba was soaked in 432 mL distilled water (eight times the weight of medicinal materials) for 30 min, boiled, and then switched to decoct it for 20 min at 100°C. The remaining medicinal materials according to the prescription were weighed and soaked in 432 mL distilled water (eight times the weight of medicinal materials) for 30 min, then added to the Ephedrae Herba decoction, which had been decocted for 20 min, and decocted together for 40 min after boiling. Subsequently, the decoction was filtered together to obtain filtrate immediately. Moreover, 342 mL distilled water was added (six times the weight of medicinal materials) to the residue and decocted for 40 min before filtering. The two filtrates were combined and concentrated under reduced pressure for lyophilization. The lyophilized powder obtained was ground and stored in a sealed bag for later use.

### 2.3. Standard Preparation

Appropriate amounts of liquiritin, glycyrrhizic acid, Schisandrol A, cinnamic acid, paeoniflorin, asarinin, and 6-gingerol were dissolved in methanol to prepare the stock solution. The stock solution was diluted to the concentration required to obtain a standard curve.

### 2.4. Xiaoqinglong Decoction (XQLT) Test Sample Preparation

The freeze-dried XQLT powder (0.4 g) was accurately weighed, dissolved in methanol (10 mL), and ultrasonicated at room temperature for 30 min. The obtained suspension was centrifuged (5000 *r*·min^−1^) for 10 min, and the supernatant was removed and filtered using a 0.22 *μ*m membrane.

### 2.5. Chromatographic Conditions

HPLC analysis was performed using an Agilent 1260 HPLC system equipped with a G1315D DAD VL detector. The chromatographic separation was performed using an Agilent Zorbax SB-C18 column (4.6 mm × 250 mm, 5 *μ*m) maintained at 40°C. The mobile phase consisted of 0.1% phosphoric acid in water (A) and acetonitrile (B) with the following optimized gradient elutions: 0 min, 5% B; 10 min, 5% B; 15 min, 10% B; 30 min, 12% B; 50 min, 15% B; 54 min, 17% B; 70 min, 22% B; 78 min, 26% B; 90 min, 28% B; 97 min, 35% B; 108 min, 49% B; 125 min, 57.9% B; and 135 min, 95% B. The flow rate was 0.8 mL·min^−1^, with an injection volume of 10 *μ*L, and the detection wavelength was set at 240 nm.

### 2.6. Experimental Animals

Forty-eight male specific pathogen-free (SPF) Wistar rats (weighing 180–200 g, 12 in each group) were purchased from Beijing Vital River Laboratory Animal Technology Co., Ltd. (experimental animal license number: Beijing SYXK [Beijing] 2016-0006). Rats were acclimatized to the environment for 1 week before the experiment. During this period, rats had free access to food and water. The animal room was maintained under 12 h light/dark cycles, with a relative humidity of 40%–60% and a temperature of 23 ± 2°C. The use of experimental animals for the study complied with the Animal Protection Committee of Shandong University of Traditional Chinese Medicine (No.: SDUTCM20200615001) requirements for laboratory animal handling and animal welfare.

### 2.7. Animal Models

**Figure 1:**
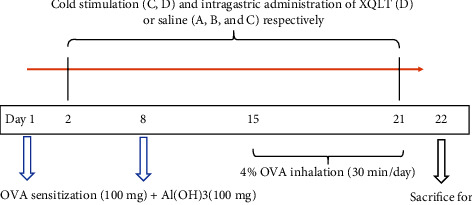
Experimental animal intervention process.

SPF Wistar rats that had been acclimated for 1 week were randomly assigned to four groups according to the schedule: Group A (control); Group B (OVA-sensitized rats); Group C (OVA-sensitized rats stimulated with cold conditions); and Group D (intervention with XQLT in OVA-sensitized rats stimulated with cold conditions). An OVA solution (1 mg·mL^−1^) was prepared by adding aluminum hydroxide gel (1%) to V-grade OVA. Rats in Groups B, C, and D received intraperitoneal injections of 100 mg OVA and 100 mg aluminum hydroxide on days 1 and 8, respectively. On the 15th day, the rats were stored in an airtight container connected to a nebulizer, and 4% OVA was added to the nebulizer to nebulize the rats for 30 min. The rats in Group A were treated with saline instead of OVA aerosol. Rats were nebulized until the end of the experiment. In groups C and D, rats were exposed to cold conditions from day 2 onward. The rats were placed in a refrigerator (0°C) for 3 h every day, followed by placing them in cold water (20 ± 2°C) until they could not swim (Figure 1) [16].

The asthma model was successfully established. The rats showed signs and symptoms of asthma, including decreased activity, arched back, shortness of breath, wheezing, easy hair loss, urinary incontinence, fecal incontinence, cold limbs, white secretions from the nose, and pale lips.

### 2.8. Experimental Design

The rats in Group D were administered XQLT freeze-dried powder (0.0694 g/100 g/d) by intragastric gavage from days 2 to 21 after swimming. The other three groups (A, B, and C) were submitted to intragastric gavage with saline solution in order to undergo the same stress as animals treated with XQLT. On day 21, the rats fasted overnight. On day 22, the rats were anesthetized by intraperitoneal injection of low-dose sodium pentobarbital (50 mg/kg) and sacrificed by cervical dislocation. Blood and tissue samples were also collected.

### 2.9. Pulmonary Function

An AniRes 2005 (BestLab, Beijing, China) lung function instrument was used to assess pulmonary function. The rats were provided only water for 24 h before testing.

### 2.10. Cytokine Analysis

Blood was collected from the abdominal aorta and placed at room temperature for 2 h and centrifuged (3500 *r*·min^−1^) for 20 min. Moreover, the supernatant was used as a serum. IL-4 and IL-13 levels were determined using a rat ELISA kit.

### 2.11. Bronchoalveolar Lavage Fluid

Two milliliters of BALF were collected and centrifuged (5000 *r*·min^−1^) at 4°C for 5 min. TNF-*α* and IFN-*γ* levels were determined using a rat ELISA kit.

### 2.12. Hematoxylin and Eosin (H&E) Staining

Paraffin sections of lung tissue specimens were prepared, deparaffinized, rehydrated, stained with hematoxylin and eosin (H&E), and examined under an optical microscope (Olympus, Tokyo, Japan) [17].

### 2.13. Transmission Electron Microscopy

Lung tissue was treated with the fixative (2% glutaraldehyde and 0.22 mmol·L^−1^ sucrose phosphate buffer) for 4 h, rinsed (0.1 M phosphoric acid) thrice (15 min), then fixed (2% osmic acid) for 2 h, and rinsed thrice. It was then dehydrated with ethanol and embedded in epoxy resin. Finally, after being stored in the oven (37°C) overnight, it was dried for 12 h (60°C). The lungs were sliced (50–100 nm) using an ultrathin sectioning machine. After double staining with uranium acetate and lead nitrate, autophagosomes were examined by transmission electron microscopy (Olympus, Tokyo, Japan).

### 2.14. Masson Staining

Deparaffinized and rehydrated lung tissue sections were obtained using the same method as described for H&E staining. Masson staining follows the kit's instructions. First, the sections were incubated with configured Weigert hematoxylin solution, differentiated with 1% acid ethanol differentiation solution, converted back to blue with Masson blue solution, and stained with Li chunhong acid fuchsin staining solution. Subsequently, the sections were incubated with a phosphomolybdic acid solution, directly dyed with an aniline blue staining solution, and rinsed with a 0.2% acetic acid solution. Finally, after the sections were dehydrated with 95% ethanol once and absolute ethanol thrice, the lung tissue sections were transparentized and sealed. Images of the lung tissue were obtained using an optical microscope (Olympus, Tokyo, Japan). The lungs of the rats in each group were analyzed using the image analysis software ImagePro Plus 6.0. Collagen fibers are blue, muscle fibers and red cells are red, and the nucleus is blue-black.

### 2.15. Immunohistochemical Studies

Anti-SQSTM1/p62 was used to measure the immune reactivity of p62. The paraffin sections of the lung tissue were prepared using the method mentioned in H&E staining, placed in an oven, baked at 55°C for 2 h, deparaffinized thrice with xylene for 10 min, rehydrated with gradient ethanol, and antigen-retrieved with citrate buffer. Subsequently, they were quenched with 3% hydrogen peroxide (10 min). They were incubated with primary antibodies overnight at 4°C, followed by the secondary antibody for 20 min at room temperature. Finally, the sections were stained with Dolichos biflorus agglutinin, counterstained with hematoxylin, dehydrated with absolute ethanol, rinsed with distilled water, transparentized with xylene, and embedded in resins. The sections were analyzed using an optical microscope (Olympus, Tokyo, Japan). ImageJ software was used to analyze the p62 expression.

### 2.16. Quantitative Real-Time Polymerase Chain Reaction (qRT-PCR)

The lung tissue stored in the refrigerator at –80°C was taken out and cut. After being extracted from the lung tissue using Trizol reagent (Invitrogen, Thermo Fisher Scientific, Inc., Waltham, MA, USA), total RNA was reverse transcribed into cDNA using the PrimeScript™ RT reagent Kit with gDNA Eraser (Perfect Real Time) kit (Takara Bio Inc., Osaka, Japan). SYBR® Premix Ex Taq™ (Takara Bio Inc., Osaka, Japan) was used for qRT-PCR to obtain quantitative information on the mRNA expression of HIF-1*α*, Beclin 1, LC3, ATG5, mTOR, and p62 in the lung tissue. HIF-1*α* is an important marker of hypoxia and a downstream target of mTOR. Under hypoxic conditions, mTOR expression is upregulated and HIF-1*α* increases p62 expression, thereby decreasing autophagy. Additionally, the transformation of LC3-I to LC3-II is a marker of autophagosome formation and Beclin 1 of autophagy. The information on primers is provided in Table 1. The 2^−ΔΔCt^ method was used for the calculation of data, which were presented as normalized expressions relative to glyceraldehyde-3-phosphate dehydrogenase mRNA.

### 2.17. Western Blotting

Total protein was extracted from the lung tissue using radio immunoprecipitation assay lysate. The protein was mixed with 2 × Laemmli sample buffer, boiled at 100°C for 5 min, denatured, separated, and transferred to a nitrocellulose membrane. After blocking with 5% nonfat milk powder at room temperature for 1 h, the membrane was incubated with primary antibodies at 4°C overnight. The secondary antibodies were diluted with a blocking solution, and the membrane was incubated with a horseradish peroxidase-conjugated secondary antibody solution for 1 h at room temperature. After each incubation, the membrane was washed with Tris Buffered Saline with Tween thrice for 5 min each. The protein band was visualized using an electrochemiluminescent reagent and exposed to X-fi film. The mean density of each protein band was measured using ImageJ software and normalized to *β*-actin content as a loading control.

### 2.18. Statistical Analyses

The Statistical Package for the Social Sciences version 13.0 was used for the analyses and the data were expressed as the mean ± standard deviation. One-way analysis of variance was used to analyze the differences between groups. A change was considered significant at *P* < 0.05 (two-tailed).

## 3. Results

### 3.1. Qualitative and Quantitative Analysis of XQLT

**Figure 2:**
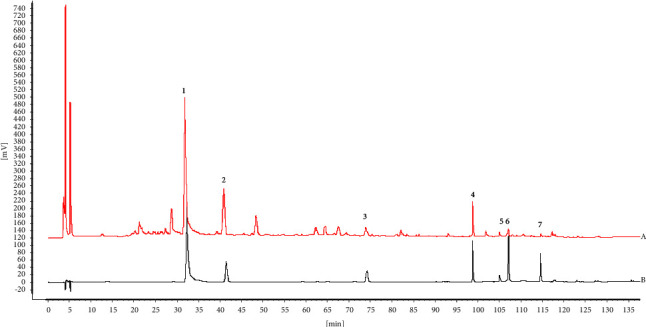
High-performance liquid chromatography fingerprint of (a) sample and (b) mixed control solutions (the detection wavelength was set at 240 nm, and the time is expressed in minutes). Paeoniflorin (1), liquiritin (2), cinnamic acid (3), glycyrrhizic acid (4), 6-gingerol (5), schisandrol A (6), and asarinin (7).

Seven peaks were identified as paeoniflorin (peak 1), liquiritin (peak 2), cinnamic acid (peak 3), glycyrrhizic acid (peak 4), 6-gingerol (peak 5), schisandrol A (peak 6), and asarinin (peak 7) and compared with the standards based on retention time (Figure 2). Standard curves and regression equations were established to quantify the seven index components in the XQLT. The concentrations of paeoniflorin, liquiritin, cinnamic acid, glycyrrhizic acid, 6-gingerol, schisandrol A, and asarinin in the lyophilized powder were 14.45, 3.85, 1.03, 3.93, 0.59, 0.24, and 0.091 mg/g, respectively.

### 3.2. Pulmonary Function

Inspiratory resistance (Ri), expiratory resistance (Re), and lung compliance (Cldyn) were used to evaluate lung function in rats. As is shown in Table 2, Ri and Re increased and Cldyn decreased on the 21^st^ day in Group B (OVA-sensitized) and C (OVA-sensitized followed by cold intervention) rats. Cold stimulation aggravated pulmonary damage in OVA-sensitized rats. Treatment with XQLT (Group D) reversed these changes. Lung function recovered in asthmatic rats. XQLT had a positive effect on asthma.

### 3.3. Enzyme-Linked Immunosorbent Assay

**Figure 3:**
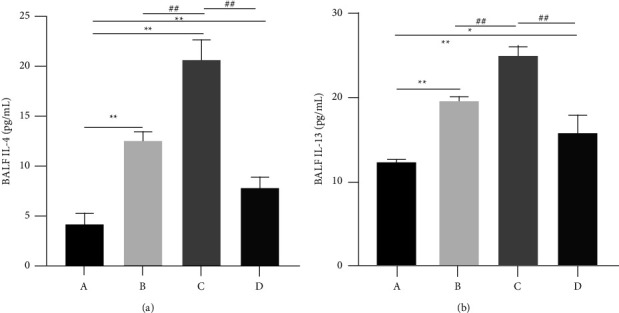
Levels of interleukin IL-4 and IL-13 in the bronchoalveolar lavage fluid of rats. (a) IL-4, (b) IL-13. Group (A) Control; Group (B) OVA-sensitized rats; Group (C) OVA-sensitized rats stimulated with cold conditions; Group (D) intervention with XQLT in OVA-sensitized stimulated with cold conditions. ^*∗*^*P* < 0.05 and ^*∗∗*^*P* < 0.01, compared with Group A; ##*P* < 0.01 compared with either Group B or C XQLT: Xiaoqinglong decoction. OVA: ovalbumin.

**Figure 4:**
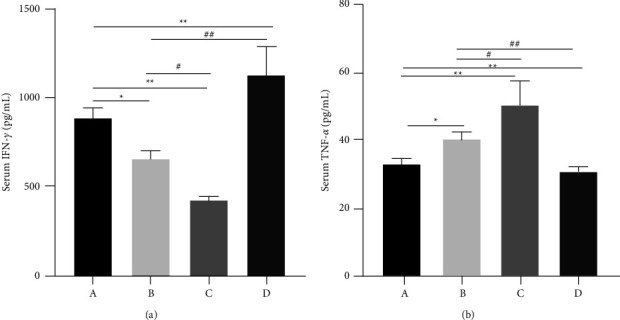
Levels of interferon-*γ* (INF-*γ*) and tumor necrosis factor (TNF)-*α* in the serum of rats. (a) INF-**γ** and (b) TNF-*α*. Group A: Control; Group B: OVA-sensitized rats; Group C: OVA-sensitized rats stimulated with cold conditions; Group D: intervention with XQLT in OVA-sensitized stimulated with cold conditions. ^*∗*^*P* < 0.05 and ^*∗∗*^*P* < 0.01, compared with Group A; #*P* < 0.05, ##*P* < 0.01, compared with either Group B or C XQLT: Xiaoqinglong decoction. OVA: ovalbumin.

The levels of IL-4 and IL-13 in the BALF are depicted in Figure 3(a) and 3(b), respectively, and of IFN-*γ* and TNF-*α* in Figure 4(a) and 4(b), respectively. The levels of IL-4 and IL-13 in the BALF were significantly (*P* < 0.01) higher in Groups B and C than in the normal group. XQLT treatment significantly (*P* < 0.01) reversed the levels of inflammatory factors. A similar trend was observed for TNF-*α* levels (Figure 4(b)). In contrast, IFN-*γ* levels significantly decreased in Groups B and C, and XQLT treatment reversed the INF- *γ* levels (Figure 4(a)).

### 3.4. H&E Staining

**Figure 5:**
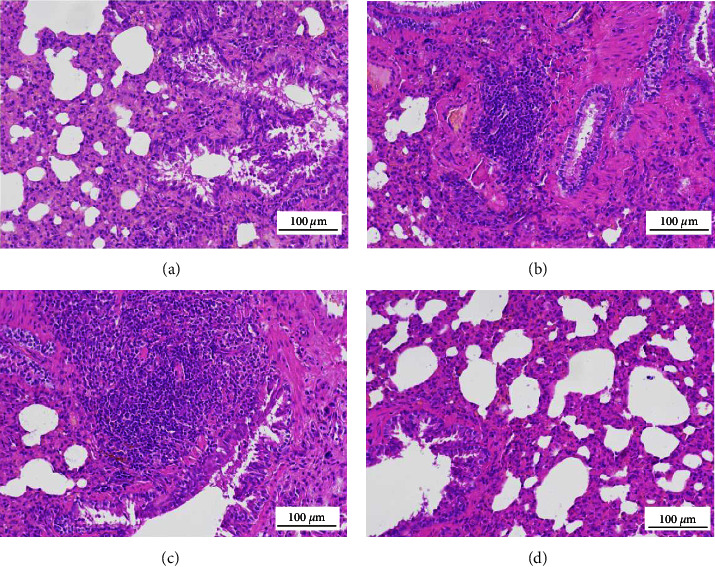
Histological features of the lung tissues observed after hematoxylin and eosin staining (200×). ((a) Group A, (b) Group B, (c) Group C, (d) Group D). Group A: Control; Group B: OVA-sensitized rats; Group C: OVA-sensitized rats stimulated with cold conditions; Group D: intervention with XQLT in OVA-sensitized stimulated with cold conditions. XQLT: Xiaoqinglong decoction. OVA: ovalbumin.

In Group A, there was no inflammatory infiltration; the size of the alveoli was normal; the bronchial structure was intact; and the cells were neatly arranged. Group B showed evident inflammatory cell infiltration around bronchial mucosa epithelial cells and alveoli. Moreover, in Group B, the bronchial structure was damaged, the hierarchy was unclear, the thickness of the epithelium in the alveoli was enhanced, and evident edema, congestion, and infiltration of inflammatory cells were observed in the alveoli. In Group C, the bronchial structure was damaged, the epithelial cells were not arranged regularly, the bronchus was infiltrated by many inflammatory cells, the airway epithelial structure was thickened, and the alveoli were filled with eosinophilic protein-like substances. The overall pathological condition was more severe in Group B than in Group A. In Group D, the bronchial structure was intact with reduced ciliary adhesions, and decreased inflammatory cell infiltration in the alveoli was observed. XQLT treatment improved the structural changes in the lung caused by OVA and OVA followed by the cold intervention (Figure 5).

### 3.5. Transmission Electron Microscopy

**Figure 6:**
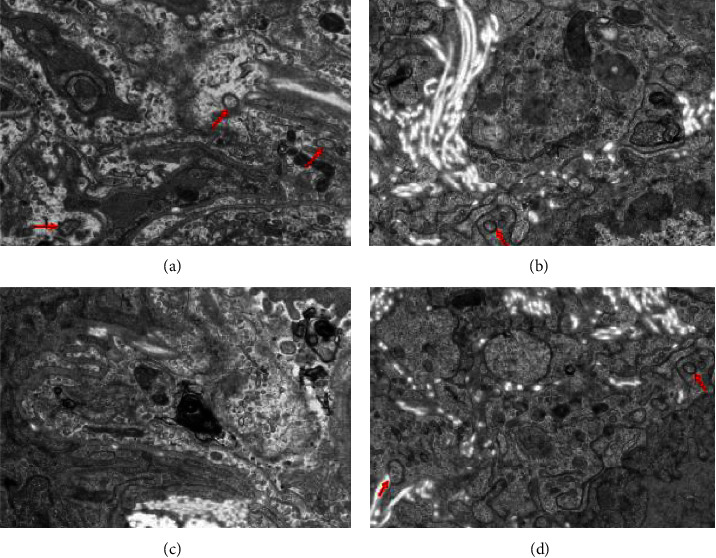
The autophagosomes (red arrow) in the lung tissues observed in transmission electron microscopy. ((a) Group A, (b) Group B, (c) Group C, (d) Group D). Group A: Control; Group B OVA-sensitized rats; Group C: OVA-sensitized rats stimulated with cold conditions; Group D: intervention with XQLT in OVA-sensitized stimulated with cold conditions. XQLT: Xiaoqinglong decoction. OVA: ovalbumin.

Transmission electron microscopy revealed that the alveolar structure of rats in Group A was evenly arranged; cytoplasm was abundant; and mitochondrial structures were normal. There were evident changes in the alveoli in Group B, including nuclear shedding, wrinkles, and nuclear membrane depression. In Group C, the tissue structure was disordered, and damaged vacuolar mitochondria were observed. The formation of autophagosomes with an evident double-layer structure was observed in Group D after the XQLT intervention (Figure 6).

### 3.6. Masson Staining

Collagen fibers appeared blue after Masson staining. The deposition of collagen fibers in the bronchi and surrounding tissues of Group B was significantly higher and the bronchial wall thicker than those of Group A. OVA + cold stimulation resulted in a large number of collagen fibers being attached to the bronchi and its surrounding tissues; thickening of the airway wall and severe airway narrowing was observed. Inflammation was further exacerbated in Group C. XQLT intervention effectively improved the deposition of collagen fibers in lung tissue and recovered the damage to the airway to a large extent.

### 3.7. Immunohistochemical Studies

p62 expression significantly increased in Group B and was further increased by cold stimulation. XQLT intervention significantly reversed the elevated expression of p62.

### 3.8. Quantitative Real-Time PCR

**Figure 7:**
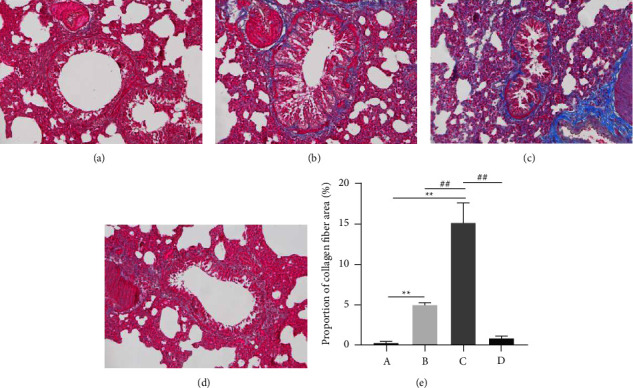
mRNA expression of HIF-1*α* (a), LC3 (b), ATG 5 (c), mTOR (d), Beclin 1, (e) and p62 (f) in the lung tissue of rats. Group A: Control; Group B: OVA-sensitized rats; Group C: OVA-sensitized rats stimulated with cold conditions; Group D: intervention with XQLT in OVA-sensitized stimulated with cold conditions. ^*∗*^*P* < 0.05 and ^*∗∗*^*P* < 0.01, compared with Group A; #*P* < 0.05, ##*P* < 0.01, compared with either Group B or C XQLT: Xiaoqinglong decoction. OVA: ovalbumin.

OVA intervention increased the expression of autophagy-related genes ATG5, Beclin 1, and LC3. Cold stimulation inhibited autophagy. XQLT intervention significantly (*P* < 0.01) increased the levels of LC3, ATG5, and Beclin 1, and mTOR expression was inhibited. After XQLT intervention, the hypoxic condition of the body was improved, and the expression of p62 decreased, which improved asthma symptoms, as shown in Figure 7.

### 3.9. Western Blotting

**Figure 8:**
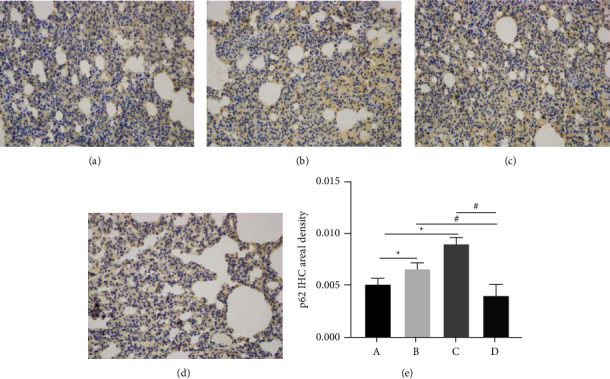
Western blotting analysis of anti—LC3 II/I and Beclin 1 protein expressions. Group A: Control; Group B: OVA-sensitized rats; Group C: OVA-sensitized rats stimulated with cold conditions; Group D: intervention with XQLT in OVA-sensitized stimulated with cold conditions. ^*∗∗*^*P* < 0.01, compared with Group A; ##*P* < 0.01, compared with either Group B or C XQLT: Xiaoqinglong decoction. OVA: ovalbumin.

The detection of LC3 expression showed that the LC3-II/I ratio was significantly enhanced after XQLT intervention (Figure 8). Beclin 1 expression was increased in OVA-sensitized rats, decreased after cold stimulation, and increased after XQLT intervention.

## 4. Discussion

**Figure 9:**
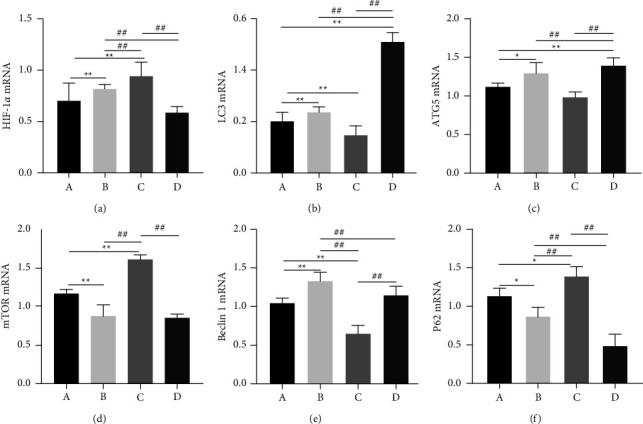
The degree of collagen fibers around the bronchus after Masson staining. (a) Group A, (b) Group B, (c) Group C, (d) Group D, e: Proportion of collagen fiber area). Group A: Control; Group B: OVA-sensitized rats; Group C: OVA-sensitized rats stimulated with cold conditions; Group D: intervention with XQLT in OVA-sensitized stimulated with cold conditions. ^*∗∗*^*P* < 0.01 compared with Group A; ##*P* < 0.01 compared with either Group B or C XQLT: Xiaoqinglong decoction. OVA: ovalbumin.

IL-13 can stimulate the excessive secretion of mucus by airway epithelial cells, leading to airway blockage [18]. IL-4 and IL-13 can enhance bronchial remodeling and recruitment of mast cells, basophils, and eosinophils [19, 20], aggravating airway inflammation in asthma. Our results indicated that OVA-sensitized rats exposed to cold stimulation increased the expressions of IL-4 and IL-13. H&E staining also showed that inflammatory infiltration in OVA-sensitized rats increased after cold stimulation (Figure 5). After XQLT intervention, asthma symptoms were alleviated, and the level of autophagy in asthmatic rats after cold stimulation was improved, which may be related to the recovery of autophagy levels by decreasing the expression of inflammatory factors and increasing the expression of IFN-*γ* [21]. Masson's staining results showed that excessive collagen deposition would lead to airway stenosis by blocking the airways. Collagen fiber content was significantly increased in the asthma model (OVA-sensitized rats). Exposure to cold conditions further increased collagen fiber content, suggesting that cold is a key factor in inducing airway inflammation (Figure 9). In the cold state, the phosphorylation level of mTOR increases, and the transformation of LC3-I to LC3-II decreases, indicating that cold has an inhibitory effect on autophagy [22]. These changes lead to abnormal metabolism *in vivo* and prevent the timely discharge of pathological products, thereby affecting the development of asthma. The results obtained in this study were consistent with those reported in the literature. Cold stimulation decreases autophagy and increases airway inflammation in OVA-sensitized asthmatic rats. Cold exposure reduced autophagosome formation in lung tissues, as observed under a transmission electron microscope.

**Figure 10:**
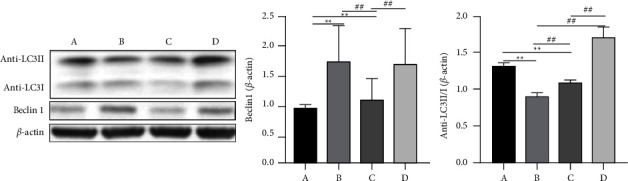
Expression of p62 in rat lung tissue. (a) Group A, (b) Group B, (c) Group C, (d) Group D, e: p62 IHC areal density). Group A: Control; Group B: OVA-sensitized rats; Group C: OVA-sensitized rats stimulated with cold conditions; Group D: intervention with XQLT in OVA-sensitized stimulated with cold conditions. ^*∗*^*P* < 0.05, compared with Group A; #*P* < 0.05, compared with either Group B or C XQLT: Xiaoqinglong decoction. OVA: ovalbumin.

LC3-II is formed by LC3-I and phosphatidylethanolamine, which is bound to autophagosomes and can induce autophagy. PCR and western blotting after XQLT intervention revealed a significant increase in LC3 II/I expression (Figure 7(b)), indicating the formation of autophagosomes in rats. The LC3 interacting region-binding domain of p62 binds to the autophagy receptor protein ATG8/LC3. After p62 recruits the ubiquitinated protein through the ubiquitin-related domain, it forms a complex with the LC3-II protein. P62 brings ubiquitin cargo to the autophagosome for selective autophagy and is dissolved together in the lysosome [23]. Immunohistochemistry (Figure 10) and PCR (Figure 7(f)) showed reduced p62 content following XQLT intervention. The reduced p62 content suggests that XQLT can promote autophagy to relieve airway inflammation.

Beclin 1 is a component of autophagosome membrane nucleation [24]. The expression of Beclin 1 and ATG5 in airway smooth muscle cells is increased in patients with asthma [25]. However, some studies have shown that ATG5 expression does not increase in the airway epithelium, airway smooth muscle cells, and inflammatory cells in asthmatic patients [26]. Therefore, the activation pattern of autophagy in the lungs of patients with asthma and its correlation with disease development remains unclear. In the OVA-induced allergic asthma mouse model, the expressions of ATG5, LC3, and Beclin 1 in the lungs and lavage fluid decreased. Simvastatin treatment reduced airway inflammation, remodeling, and hyperreactivity. It reduced IL-4, IL-5, and IL-13 levels in the BALF; increased the expression of autophagy genes ATG5, LC3II, and Beclin 1 in the lungs; and enhanced autophagy function [27]. In addition, the loss of autophagy in airway epithelial cells leads to the swelling of bronchial epithelial cells, resulting in high airway responsiveness and accumulation of increased p62 [28]. These findings indicate that the regulation of autophagy can also play a therapeutic role in asthma, but its level may need to be controlled within a certain range. However, the activation of autophagy under inflammatory conditions leads to non-caspase-dependent cell death of eosinophils and neutrophils. The potential protective effect of autophagy on activated granulocytes has been discussed [29], further demonstrating the positive role of autophagy in diseases.

In this study, the level of autophagy was increased in the OVA-sensitized rats, which decreased after cold stimulation. The level of autophagy was increased after XQLT intervention in rats, and asthma symptoms were also eased, explaining the positive role of autophagy in asthma pathogenesis. Although the onset of asthma is mostly seasonal and a cold is one of the triggers of asthma, the autophagy response to environmental stimuli, cell types, and activation states is usually diverse. Therefore, autophagy also requires close examination [29]. Simultaneously, the effect of autophagy on asthma should be carefully evaluated. This study only evaluated the changes in the autophagy level, and its mechanism remains unclear. We should further explore the damaging or protective effects of autophagy on asthma. As a compound medicine, the pharmacological components and compounds of XQLT need to be further explored to elucidate its molecular mechanisms of action.

## 5. Conclusions

By observing the inflammatory state of the lung tissue and the expression of autophagy-related proteins in rats, it has been shown that cold is one of the key environmental factors that induces asthma, which can lead to a decrease in autophagy. This phenomenon may be related to the reduction in cell metabolism, insufficient adenosine triphosphate production, and decreased LC3 transcriptional activity. However, the related mechanisms require further study. After treatment with XQLT, the autophagy level increased, lung inflammation decreased, and asthma was relieved. This experimental result provides a new idea for the clinical treatment of cold-induced asthma.

## Figures and Tables

**Table 1:** List of primer sequences.

Gene	Forward primer	Reverse primer
GAPDH	ACAGCAACAGGGTGGTGGAC	TTTGAGGGTGCAGCGAACTT
HIF-1*α*	AGGGGAAAGAACAAAACACGCA	TTTCTTGTAGCCACACTGCGG
Beclin 1	GTCTAAGGCGTCCAGCAGCAC	CGCCTGGGCTGTGGTAAGTAA
LC3	ATCAACATTCTGACGGAGCGG	TGGATTTCTTCAGTTGCTTGGC
ATG5	GCCATACTATTTGCTTTTGCCA	ATTTCAGGGGTGTGCCTTCAT
mTOR	TGGAGAACCAGCCCATAAGAAA	TGAGAGAAATCCCGACCAGTGA
P62	CCTGTCAAGCAGTATCCAAAGTT	CCTTGGCTTTGTCTCTCATCG

**Table 2:** The pulmonary function in different groups of rats (*n* = 6).

Groups	Ri (cm H_2_O·s·mL^−1^)		Re (cm H_2_O·s·mL^−1^)		Cldyn (mL·cm^−1^ H_2_O)	
	Baseline	Day 21	Baseline	Day 21	Baseline	Day 21
Group A	1.917 ± 0.192	1.136 ± 0.075	1.945 ± 0.176	1.504 ± 0.025	0.228 ± 0.032	0.232 ± 0.020
Group B	1.924 ± 0.205	2.908 ± 0.344^*∗∗*^	1.820 ± 0.190	2.972 ± 0.285^*∗∗*^	0.213 ± 0.038	0.202 ± 0.028
Group C	1.763 ± 0.262	3.322 ± 0.060^*∗∗*^^##^	1.900 ± 0.256	3.720 ± 0.751^*∗∗*^^#^	0.226 ± 0.035	0.026 ± 0.000^*∗∗*^^##^
Group D	1.906 ± 0.147	2.907 ± 0.243^ΔΔ^	1.879 ± 0.226	2.879 ± 0.294^ΔΔ^	0.213 ± 0.026	0.223 ± 0.064^ΔΔ^

Group A, Control; Group B, OVA-sensitized rats; Group C, OVA-sensitized rats stimulated with cold conditions; Group D, intervention with XQLT in OVA-sensitized stimulated with cold conditions. ^*∗∗*^*P* < 0.01 compared with Group A. ##*P* < 0.01 compared with Group B. ΔΔ*P* < 0.01compared with Group C. XQLT, Xiaoqinglong decoction; OVA, ovalbumin.

## Data Availability

All data generated or analyzed during this study are included in this published article.
